# New therapeutic perspectives – amyloid removal

**DOI:** 10.1186/1750-1172-10-S1-I17

**Published:** 2015-11-02

**Authors:** Mark Pepys

**Affiliations:** 1 FRS, Wolfson Drug Discovery Unit & NHS National Amyloidosis Centre, Centre for Amyloidosis & Acute Phase Proteins, University College London & Royal Free Hospital, London, UK

## Background

In systemic amyloidosis, disease is caused by extracellular accumulation of amyloid fibrils which, unlike other interstitial debris, are not cleared and which disrupt tissue structure and function. Direct removal of amyloid deposits is required to preserve and possibly restore tissue and organ function. We are targeting serum amyloid P component (SAP) for this purpose. SAP normal plasma protein is a normal plasma protein which binds to all amyloid fibrils and is thus always present in all human amyloid deposits. Administration of hexanoyl bis(D–proline) (CPHPC) swiftly depletes circulating SAP but leaves some SAP in amyloid deposits as an amyloid-specific antigen target. In human SAP transgenic mice with systemic AA amyloidosis, CPHPC treatment, to deplete human SAP from the plasma, followed by a single dose of anti-human SAP antibodies produced swift, almost complete, clearance of visceral amyloid (Bodin *et al*. *Nature*, 2010;468:93-7). In a mouse SAA transgenic systemic amyloidosis model, which uniquely includes cardiac amyloid, a second dose of anti–SAP antibody, after the first dose had eliminated massive liver and spleen deposits, significantly removed amyloid from the heart (Simons *et al*. *Proc Natl Acad Sci USA*. 2013;110:16115-20). Amyloid clearance by anti-SAP antibody required classical complement pathway activation and macrophages. Amyloid destruction was mediated by multinucleated giant cells (MGCs), formed by macrophage fusion. MGCs have abundant surface membrane ruffles, enabling the engulfment of very large complement opsonised amyloid targets which were then swiftly destroyed within phagolysosomes. Amyloid clearance was maximal by 14 days. No ill effects were detected. After licensing this new treatment in February 2009, GlaxoSmithKline (GSK) fully humanised our optimal mouse monoclonal anti-SAP antibody and prepared for the first in human clinical study that started in June 2013. We have lately reported that a single dose of humanized monoclonal anti-SAP antibody, following depletion of circulating SAP by CPHPC, substantially reduced the amyloid load, especially from the liver, in patients with systemic AL, AA and AApoAI amyloidosis, (Richards *et al*, *New Engl J Med*, July 15 2015; DOI: 10.1056/NEJMoa1504942).

## Methods

GSK’s phase I study of the obligate therapeutic partnership of CPHPC and anti–SAP antibody in patients with systemic amyloidosis is ongoing (http://www.clinicaltrials.gov/ct2/show/NCT01777243?term=amyloid+gsk&rank=2). The first patients with ATTR are about to be treated.

## Results

Efficacy in amyloid removal requires a sufficient dose of antibody and is associated with a transient early acute phase response of CRP and SAA, transient early mild neutrophilia and then notable depletion of plasma C3 and a less marked fall in C4 and CH50 (Richards *et al*, *New Engl J Med*, July 15 2015). Amyloid removal has not been associated with detectable additional organ dysfunction. On the contrary, abnormal liver function tests improved following clearance of hepatic amyloid and the treatment has so far been generally well tolerated (Richards *et al*, *New Engl J Med*, July 15 2015).

## Conclusions

Treatment with CPHPC and anti–SAP antibody promotes amyloid removal in all types of systemic amyloidosis tested so far. Efficacy in ATTR, in which hepatic amyloid is never present and the main organs involved are the nerves and heart, will be of considerable interest.

The Phase I study of CPHPC and anti-SAP antibody is funded by GlaxoSmithKline.

